# BacMam virus-based surface display for HCV E2 glycoprotein induces strong cross-neutralizing antibodies and cellular immune responses in vaccinated mice

**DOI:** 10.1186/s13027-021-00407-x

**Published:** 2021-12-18

**Authors:** Ebrahim Kord, Farzin Roohvand, Jean Dubuisson, Thibaut Vausselin, Hosein Nasr Azadani, Abolfazl Keshavarz, Ahmad Nejati, Katayoun Samimi-Rad

**Affiliations:** 1 Department of Virology, School of Public Health, Tehran University of Medical Sciences, Enqelab Square, P.O. Box 1417613151, Tehran, Iran; 2 Infectious Diseases and Tropical Medicine Research Center, Resistant Tuberculosis Institute, Zahedan University of Medical Sciences, Zahedan, Iran; 3 Department of Virology, Pasteur Institute of Iran (IPI), No. 69, Pasteur Ave, P.O. Box 1316943551, Tehran, Iran; 4 CNRS, Inserm, CHU Lille, Institut Pasteur de Lille, Batiment, IBL, CS50477, Molecular & Cellular Virology, U1019 - UMR 8204 - CIIL- Center for Infection and Immunity of Lille, University Lille, 59021 Lille Cedex, France

**Keywords:** HCV, gpE2, Baculovirus, BacMam virus, Surface display, Vaccine

## Abstract

**Background:**

Despite recent advancements, limitations in the treatment and control of hepatitis C virus (HCV) infection reprioritized the studies for invention of an efficient HCV vaccine to elicit strong neutralizing antibodies (NAbs) and cellular responses.

**Methods:**

Herein, we report molecular construction of a BacMam virus-based surface display for a subtype-1a HCV gpE2 (Bac-CMV-E2-gp64; Bac) that both expressed and displayed gpE2 in mammalian cells and bacouloviral envelope, respectively.

**Results:**

Assessments by western blotting, Immunofluorescence and Immunogold-electron microscopy indicated the proper expression and incorporation in insect cell and baculovirus envelope, respectively. Mice immunized in three different prime-boost immunization groups of: Bac/Bac, Bac/Pro (bacoulovirus-derived gpE2) and Bac/DNA (plasmid DNA (pCDNA)-encoding gpE2) developed high levels of IgG and IFN-γ (highest for Bac/Bac group) indicating the induction of both humeral and cellular immune responses. Calculation of the IgG2a/IgG1 and IFN-γ/IL-4 ratios indicated a Th1 polarization of immune responses in the Bac/Bac and Bac/DNA groups but a balanced Th1-Th2 phenotype in the Bac/Pro group. Sera of the mice in the Bac/Bac group provided the highest percentage of cross-NAbs against a subtype-2a HCVcc (JFH1) compared to Bac/Pro and Bac/DNA groups (62% versus 41% and 6%).

**Conclusions:**

Results indicated that BacMam virus-based surface display for gpE2 might act as both subunit and DNA vaccine and offers a promising strategy for development of HCV vaccine for concurrent induction of strong humoral and cellular immune responses.

**Supplementary Information:**

The online version contains supplementary material available at 10.1186/s13027-021-00407-x.

## Background

Around 71 million people worldwide are suffering from chronic hepatitis C virus (HCV) infection that results to sever liver diseases or malignancies in majority, while the burden of the disease is expanding by millions of newly infected cases annually [1, 2]. Availability of direct-acting antivirals (DAAs) has considerably improved the treatment of patients with chronic HCV infection in recent years, but high cost, emergence of drug resistance variants, progression of the liver disease despite therapy and other shortcomings of DAAs underscored the need for development of a vaccine against HCV infection [3, 4].

The RNA genome of HCV encodes for three structural proteins including capsid and envelope (E) 1 and 2 glycoproteins (gpE1/gpE2), as well as several non-structural proteins [5]. Despite extensive studies for development of a HCV vaccine by use of HCV proteins in various antigen formulations and modalities, no approved vaccine for human use is available to date. In fact, results of the studies indicated that for being effective, an HCV vaccine needs to elicit strong neutralizing antibodies (NAbs) and potent T-cell responses [6, 7]. Of note, recognized NAbs to HCV are only raised against envelope proteins and in specific gpE2 (and much lesser extent against gpE1) and despite high variability of gpE2 among HCV genotypes, development of cross-genotype reactive NAbs by its immunization in chimpanzees is reported [8, 9]. In addition, HCV gpE2 harbors several dominant T-cell epitopes for induction of strong cellular responses that further endorse it as a candidate vaccine antigen [10, 11]. Indeed, formulation of recombinant HCV gpE1/gpE2 with human compatible adjuvant, MF59, is among few HCV vaccine candidates evaluated in human clinical trials with proper safety and encouraging potential outcomes [12]. Therefore, search for better adjuvants and vaccine platforms/modalities for induction of broad spectrum NAbs and cellular responses were undertaken [6].

In recent years among vaccine platforms, viral vectors such as adenovirus or modified vaccinia virus Ankara (MVA), attracted more interest for delivery of heterologous antigens. This was partly due to favorable safety profile, presenting the target antigen(s) in their native conformation and high level of antigen expression that resulted to stronger immune responses [13]. However, presence of pre-existing anti-vector antibodies/immunity and the limitations for the size of the inserted antigen within the genome, enhanced the search for nonhuman viral vectors that might overcome these short comings. Among studied viral vectors, insect cell-infecting baculoviruses, especially *Autographa californica multicapsid nucleopolyhedrovirus* (AcMNPV) which is a nonpathogenic and non-replicative virus in humans properly addressed the concerns and thus were utilized as both an expression system for recombinant proteins/antigens and a gene delivery vector [14, 15]. The utility of baculovirus efficient gene delivery to mammalian cells was dependent on the use of mammalian promoters such as cytomegalovirus (CMV) promoter, a technology known as “BacMam” [16]. In addition, presence of CpG motifs within the baculoviral DNA genome shows strong adjuvant properties on induction of immune responses in mammalian cells for production of pro-inflammatory cytokines [17]. This adjuvant property together with the availability of “Baculovirus Surface Display systems” for antigen presentation provided a robust and versatile vaccine manufacturing platform [18, 19]. Further improvements on this vaccine manufacturing platform was achieved by combination of “BacMam” and “Baculovirus Surface Display systems” through utilization of dual promoters consisting of baculovirus-derived (e.g.: polyhedron) and mammalian-derived (e.g.: CMV) promoters that permitted both display of the target antigens on the viral envelope and their concurrent expression upon transduction in mammalian cells. Such dual functioning (expression/display) mimic the potential advantages of both DNA and subunit vaccines for presentation of the antigens via the MHCI and MHCII pathways to elicit strong Th1/Th2 mediated induction of humoral and cell-mediated immune responses [19]. This so called “BacMam virus-based surface display” or “Baculovirus Dual Expression System” has shown strong immune responses in vaccine designs for several infectious agents including: Avian Influenza [20], *Plasmodium vivax* [21], porcine reproductive and respiratory syndrome (PRRS) [22], infectious bronchitis virus (IBV) [23], H5N1avian influenza [24] and respiratory syncytial virus (RSV) [25].

To date, most of the vaccine studies addressing the use of bacoulovirus vector have sought their fate in veterinary applications. This was mainly due to the lack of safety guidelines for their application in human immunization. However, recent declaration of the European Commission’s Health & Consumer Protection Directorate-General concerning their general safety on human health and studies on development of baculovirus-based vaccines with high safety profile had great impact on their potential applications in human immunization [26]. In this context, several pre-clinical studies in animal models have recently addressed baculovirus-based vaccines against important human infections such as influenza and rabies [19].

Although application of baculovirus and insect cells for expression of properly folded HCV gpE1/gpE2 for immunization studies was reported since late 1990s [27], but only few prior studies addressed the use of baculovirus vector for expression of HCV gpE2 which was restricted to a report on transduction and expression efficiencies of this system in hepatocytes [28], comparison of immunogenicity of E2 proteins expressed in mammalian and insect cells [29] or immunization of mice to evaluate baculovirus system for gene therapy purposes [30]. Indeed, the latter study intended to evaluate the gene therapy values of baculovirus system based on the fact that immunodeficient cells, such as HCV-infected cells, might be more selectively eliminated by recombinant baculovirus than normal cells [14]. Therefore, considering the intrinsic capability of “Baculovirus Surface Display systems” in induction of strong humoral and cellular responses, in this study, we constructed a “BacMam virus-based surface display” for both expression and surface display of HCV gpE2 and evaluated its potential as a candidate vaccine by homologous and heterologous prime–boost immunization with E2-protein/ DNA in mice model.

## Materials and methods

### Construction of plasmids and production of the recombinant baculoviruses

To generate the “BacMam virus-based surface display” for both expression and surface display of HCV gpE2 in insect cells, first a DNA fragment was synthesized that encoded in tandem for: immediate early cytomegalovirus promoter (PCMV-IE), the signal sequence of gp64 (the major envelope fusion glycoprotein of baculovirus) (SP), a stretch of six histidine residues (His6 tag), the ectodomain of HCV E2 protein (E2, aa 384-661, genotype 1a, Gen Bank accession no: AF011753.1) and the transmembrane (TM) and the cytoplasmic terminal domain (CTD) of gp64. The synthesized fragment (pCMV-SP-E2-TM-CTD) was inserted into the *Xho* I and *Not* I sites of pBluescript II SK (+) to construct the pBluescript-CMV-E2-gp64 plasmid (Biomatik Corporation, Canada). Subsequently, the synthetized fragment was excised from pBluescript-CMV-E2-gp64 and subcloned between *Xba* I and *Hind* III sites of the transfer vector pFastBac 1 (Invitrogen, Carlsbad, CA, USA) to generate pFast-CMV-E2-gp64. In order to integrate the construct into the baculovirus genome through site specific transposition and generate the corresponding recombinant bacmids, the recombinant pFast-CMV-E2-gp64 and non-recombinant pFastBac 1 as a negative control were individually transformed into the competent DH10Bac™ *E. coli* cells. The resulting recombinant bacmids were isolated from white colonies after two rounds of blue/white colony selection according to the standard procedure (Bac-To-Bac system, Invitrogen). Presence of the inserted gene was confirmed by PCR using insert-specific (Bac-E2-F and Bac-E2-R) and universal M13 (pUC/M13-F and pUC/M13-R) primers. The former primers were designed with Oligo 7 Primer Analysis Software (Molecular Biology Insights, Inc.) and the latter primers were sugessted in the Bac-To-Bac manual (Additional file 1: Table S1). The recombinant (Bac-CMV-E2-gp64) and wild type (Bac-WT) baculoviruses were generated in *Spodoptera frugiperda* (Sf9) cell lines (ATCC, USA) using the Bac-To-Bac Baculovirus Expression System (Invitrogen, Carlsbad, CA, USA) according to the manufacturer protocols. Briefly, the Sf9 cells were cultured in 6-well plate and allowed to attach for 1 h. Then, 2 µg of each recombinant bacmid DNA was mixed with 8 µl cellfectin II (Invitrogen, USA) in Grace’s Insect Cell Culture Medium, and transfected into the attached Sf9 cells. The supernatant containing released virus particles harvested 96 h post transfection, centrifuged, and used for more amplification of the viruses. For large-scale production, Sf9 cells were infected at a multiplicity of infection (MOI) of 0.2 and incubated. Five days post infection, the supernatant was centrifuged and viral particles were purified by two rounds of sucrose gradient ultracentrifugation as described previously [31]. The infectious titers of the recombinant bacoloviruses were determined by plaque assay. The viral stocks were stored at 4 °C in dark. The Bac-to-Bac system was also used to generate the recombinant baculovirus expressing HCV E2 ectodomain (Bac-E2/exp). The purified Bacolouvirus-derived rE2 protein was used as boosting immunogen in mice immunization procedures where indicated and as coating antigen for ELISA and cytokine assays. The detailed steps for generation of the recombinant Bac-E2/exp are described in the Additional file 1: Fig. S1.

The pBluescript-CMV-E2-gp64 plasmid was also used as template to construct the pEGFP-E2-NT (gp96) for evaluation of the expression of gpE2 in mammalian cells by IF microscopy (Additional file 1: Fig. S2) and pcDNA-E2-NT(gp96) plasmid encoding for the HCV gpE2 ectodomain and N-terminal domain of heat shock protein gp96 (NT(gp96)) (Additional file 1: Fig. S3). The gp96 protein is a member of Heat-Shock Proteins (HSPs) family, which has been used as a genetic adjuvant in some experiments [32]. Studies have shown that gp96 and/or its N-terminal domain stimulates the immune system by interacting with Toll-like receptors (TLRs) to activate macrophages and dendritic cells. It also stimulates T-cells by cross-presentation of peptides to MHC class I and class II molecules [33]. The pcDNA-E2-NT(gp96) plasmid was used as boosting immunogen in mice immunization procedures where indicated. All the cloning procedures were based on standard protocols [34].

### SDS-PAGE and western blot analysis

For detection of expressed E2, Sf9 cells were infected with recombinant baculovirus Bac-CMV-E2-gp64 or Bac-WT as a negative control. Three days post infection, cells supernatants (5 µl) were mixed with 2 × SDS-PAGE sample buffer, boiled and loaded to a 10% SDS-PAGE based on standard protocols [34]. For western blot analysis, 20 μl of supernatants were loaded on SDS-PAGE and the proteins were transferred to polyvinylidene difluoride (PVDF) membranes. Then, it was incubated with a mouse anti-His monoclonal antibody (1:15,000; QIAGEN, Valencia, CA) as the primary antibody. The secondary antibody was horseradish peroxidase (HRP) conjugated goat anti-mouse IgG antibody (1:3000; Southern Biotech, Birmingham, AL). Reactive bands were visualized using an enhanced chemiluminescence (ECL) Kit (Clarity™ Western ECL Substrates, Bio Rad) and light emission was detected by exposing the membrane to a Hyper film ECL (GE-Amersham).

### Indirect immunofluorescence assays (IFA)

The Sf9 cells were cultured on sterile cover slips (Nunc, Denmark) (placed in 6-well plates) and infected with the recombinant baculovirus Bac-CMV-E2-gp64 at a MOI of 0.5. Three days post infection, the infected and uninfected cells (negative control) were fixed with 4% paraformaldehyde (PFA) for 15 min at room temperature, washed and blocked with 1% bovine serum albumin for 45 min at 37 °C. Then, the cells were incubated with the primary mouse anti-His monoclonal antibody (MAb) (1:300) for 1 h at room temperature. After several washing steps, the cells were incubated with the secondary FITC-conjugated anti-mouse IgG antibody (1:100; Dako, Denmark) for 45 min at room temperature. The fluorescence signals were detected with an inverted fluorescence microscope (Olympus, UK) and the fluorescence images were captured by a digital imaging system (Nikon).

### Immunogold electron microscopy

The carbon-coated grids (Sigma Aldrich, St Louis, MO) were floated on 10 µl of purified baculovirus solution for 30 min, and blocked with 5% bovine serum albumin (BSA) in PBS. After three times washing, the grids were exposed to mouse anti-His MAb (1:100 dilution) for 45 min. Subsequently, the grids were washed and exposed to goat anti-mouse IgG conjugated with 10 nm gold particles (1:50 dilution, Sigma, USA) for 30 min. After several washing steps, the grids were stained with 1% uranyl acetate and examined under the transmission electron microscope (Zeiss LEO 906, Germany).

### Mice immunization

Three different prime-boost regimens were carried out in four groups of 6-week-old female BALB/c mice (n = 8 per group). Mice (purchased from Laboratory Animals Center, Pasteur Institute of Iran) were kept in plastic cages with standard rodent pellet and appropriate water in a centralized air conditioned facility under a constant 12:12 h light-dark cycle at room temperature and 55–60% relative humidity. Three groups of mice were immunized two times at week 0 and 3 with 1 × 10^8^ PFU purified recombinant baculoviruses (Bac-CMV-E2-gp64). At week 6 the Bac/Bac group was boosted with the same antigen, whereas the two other groups Bac/Pro and Bac/DNA were boosted with either 5 μg of rE2 protein mixed with Montanide ISA 720 (water-in-oil emulsion: Seppic, France) as adjuvant or 50 μg of pcDNA-E2-NT(gp96) plasmid. The last group (PBS) received 100 μl of PBS as a control in the same immunization periods (Table 1). All groups were vaccinated intramuscularly. Blood samples were taken from the orbital plexus of each mouse before immunization and two weeks after the last immunization (week 8) for ELISA and the neutralization assay.

**Table 1:** Immunization schedule in different mice groups

Groups	Immunization 1, 2	Immunization 3
	Week 0, 3	Week 6
Bac/Bac	Bac-CMV-E2-gp64	Bac-CMV-E2-gp64
Bac/Pro	Bac-CMV-E2-gp64	E2 protein, Montanide ISA 720
Bac/DNA	Bac-CMV-E2-gp64	pcDNA-E2-NT(gp96)
PBS	PBS	PBS

### Enzyme linked immunosorbent assay (ELISA)

The level of the E2-specific total IgG and isotypes (IgG1 and IgG2a) of the immunized mice were measured by ELISA [32]. The ratio of IgG2a/IgG1 isotype responses was also determined. Briefly, ninety-six-well maxisorb plates (Greiner, Germany) were coated with 100 µl (1 µg/ml in PBS) of the rE2 protein at 4 °C overnight, washed and blocked with 5% skim milk for 2 h at 37 °C. After washing steps, 100 µl of pooled sera (1:500) of each group was added to the plate in duplicates and incubated for 2 h at 37 °C. The wells were washed and 100 µl of the HRP-conjugated goat anti-mouse IgG, IgG1 or IgG2a (1:3000; Southern Biotech, Canada, AL) was added and incubated for another 2 h at 37 °C. Following the last washing steps, the color was developed by adding 100 µl of TMB substrate (3,3′,5,5′-tetramethylbenzidine, Sigma) to each well and incubated in a dark room. The color development was stopped by adding 50 µl sulphuric acid (2 M). The absorbance (OD) was measured at 450 nm by microplate reader (Biochrom Anthos 2020 microplate reader, UK).

### Cytokine assay

Six mice of each group were sacrificed two weeks after the last immunization. Their spleens were homogenized using a tissue grinder. Erythrocytes were lysed using ammonium-chloride-potassium (ACK) lysis buffer (NH4Cl 0.15 mM, KHCO3 1 mM and Na2EDTA 0.1 mM: pH 7.2) and washed in Dulbecco's Modified Eagle's medium (DMEM, Gibco). Single splenocyte suspensions were seeded in 96-well plates at 2 × 10^6^ cells/well in DMEM containing 10% heat-inactivated fetal bovine serum (FBS). Then, cells were stimulated with 10 µg/ml rE2 protein, 5 µg/ml concanavalin A (positive control; Sigma-Aldrich, Germany) or medium alone (negative control). The supernatants of splenocyte cultures were harvested after 72 h for IL-4 assay and 96 h for IFN-γ assay. The cytokine levels were measured using a sandwich ELISA according to the manufacturer manual (R&D, Quantikine®ELISA, USA). All assays were performed in duplicates. The optical density (OD) was measured at 450 nm ELISA reader (Biochrom Anthos 2020 microplate reader, UK) and the concentration was calculated according to standard curve.

### Neutralization assay

The plasmid pJFH-1 containing the full length cDNA of JFH-1 strain (genotype 2a; Gen Bank accession no. AB047639), kindly provided by T.Wakita was used to generate RNA as previously described [35]. In vitro transcribed RNA was electroporated into Huh-7 cells and 1 week later, extracellular virus was collected by harvesting cell culture supernatants. Viral infectivity was evaluated by infecting Huh-7 cells with serially dilutions of viral supernatant. The viral titer was determined at 3 days post-infection by a focus-forming unit (FFU) staining assay [36]. For neutralization assay, the positive control antibody containing anti-E1E2 MAbs AR5A (kindly provided by M.Law [37] (Scripps Research institute, La Jolla, CA)) was used at 10 μg/ml and immune or pre-immune sera were mixed with viral medium at 1:100 and incubated for 1 h at 37 °C. After 1 h incubation, the virus (150 FFU)-serum mixture was transferred to Huh-7 cells seeded in 96-well plates (1 × 10^4^ cells per well) for 6 h at 37 °C. At this time, the viral medium was removed and the cells further incubated for 44 h in fresh complete cell medium. Cells were then fixed and immunostained by using anti-NS5A MAb 9E10. The neutralization effects were assessed by titration and the results were shown as the percent neutralization of virus infection compared to the pre-immune sera. The assay was performed in triplicate and the results were presented as mean values.

### Statistical analysis

Statistics were performed using GraphPad Prism 7.03 for windows (Graphpad Software Inc. 2017, La jolla, California, USA). All data were analyzed by one-way ANOVA (Multiple-comprison HSD-Tukey test) and where required by Student’s t-test. *P* values less than 0.05 were considered significant. The results were expressed as mean ± SD.

## Results

### Confirmation of the generated recombinant baculovirus

**Fig. 1:**
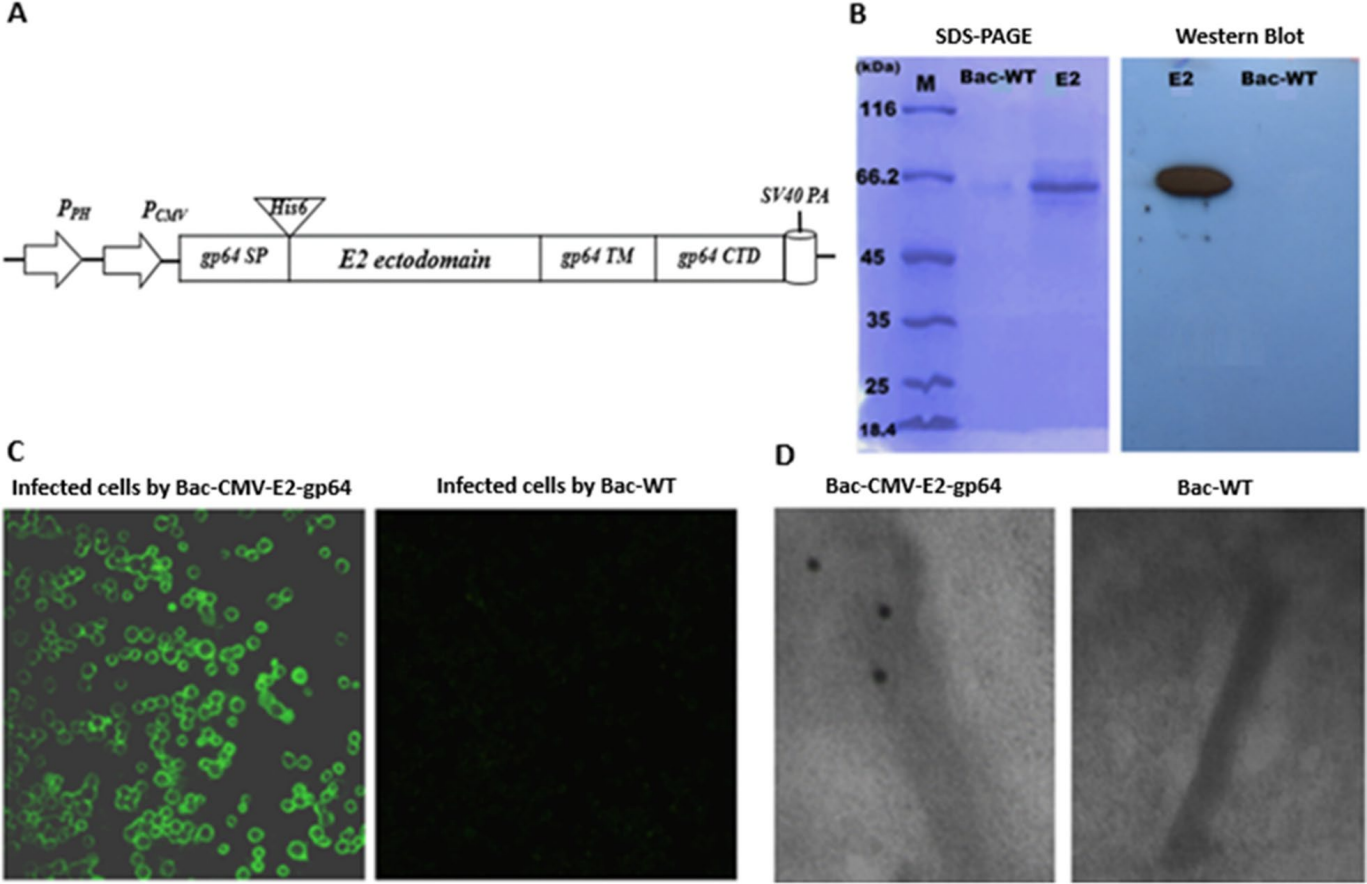
Construction of recombinant baculoviruses, and E2 protein expression and display. **a** Schematic representation of the constructed Bac-CMV-E2-gp64. P_PH_, polyhedrin promoter of baculovirus; pCMV, cytomegalovirus immediate early promoter/enhancer; gp64 SP, gp64 signal peptide; His6 tag, a stretch of six histidine residues; E2 ectodomain, HCV E2 ectodomain gene; gp64 TM, gp64 transmembrane; gp64 CTD, gp64 cytoplasmic domain; SV40PA, Simian virus 40 Poly A. **b** Expression of E2 protein in SF9 cells. The cells were infected with recombinant Bac-CMV-E2-gp64 or wild type baculovirus (Bac-WT) as a negative control. Three days post infection, the infected cells supernatants were separated on 10% SDS-PAGE gel, and subjected to western blot analysis using anti-His MAb and goat anti-mouse IgG antibodies. E2 protein is visible in a 64 kDa band, while no band was observed for Bac-WT. **c** Immunofluorescence microscopy of SF9 cells infected with recombinant baculovirus Bac-CMV-E2-gp64. The E2 protein was observed to be located on plasma membrane of cells infected by Bac-CMV-E2-gp64 (left) while no signal was detected in uninfected cells (right). **d** Display of the recombinant E2 protein on the baculoviral envelope. Immunogold electron micrograph of the purified baculoviruses Bac-CMV-E2-gp64 and Bac-WT was performed using mouse anti-His MAb as the primary antibody and anti-mouse IgG conjugated with 10 nm gold particles as the secondary antibody. Gold particles on the envelope of baculovirus Bac-CMV-E2-gp64 were detected (left) whereas no gold particles were observed on envelope of Bac-WT (right)

The constructed recombinant baculovirus (Bac-CMV-E2-gp64; Bac) for simultaneous expression/display of the HCV E2 protein consisted of pCMV-IE, gp64 SP, His6 tag, E2 ectodomain, gp64 TM and CTD under the control of polyhedron promoter (P_PH_) (Fig. 1a). Expression of E2 under the control of polyhedron promoter in the insect cells was supposed to direct the translocation into the plasma membrane and final incorporation of E2 protein into the viral envelope via the sequential actions of gp64 SP, TM and CTD. Simultaneously, Bac-CMV-E2-gp64 was also supposed to support the expression of E2 protein in the mammalian cells under the control of CMV-IE-promoter.

To examine the expression of E2 protein, the Sf9 cells were infected with the Bac-CMV-E2-gp64 or Bac-WT as a negative control. The supernatant was analyzed by western blot at 3 days post infection which indicated the expression of E2 protein with approximately 64 KDa in cells infected with Bac-CMV-E2-gp64 and not in Bac-WT infected cells (Fig. 1b). These tests were performed only with a qualitative aim to prove the expression of E2 protein by the Bac-CMV-E2-gp64 (i.e.: not a quantitative aim to estimate the yield of expression). As shown in Fig. 1c, translocation of the E2 protein to the Sf9 cells membrane was confirmed by the indirect immunofluorescence assay via detection of the fluorescence signals on the surface of the Bac-CMV-E2-gp64 infected cells. No fluorescence signal was observed in uninfected cells. To confirm whether the anchored E2 protein in the cell plasma membrane was displayed on the baculovirus envelope, purified viral particles were analyzed by immunoelectron microscopy using anti-His6 and 10 nm gold-conjugated secondary antibody. As shown in Fig. 1d, gold particles were detected on the viral envelope of Bac-CMV-E2-gp64, revealing the successful display of the E2 on the viral envelope, whereas no gold particles were observed in Bac-WT.

To evaluate the in vitro expression of E2-NT(gp96) construct by detection of GFP fluorescence, Cos-7 cells were separately transfected with pEGFP-E2-NT(gp96), pEGFP-N3 (positive control) and pcDNA3.1 (negative control) vectors. Fluorescence microscopy revealed the proper expression of the EGFP by pEGFP-E2-NT(gp96) vector compared to the pEGFP-N3 as positive control in Cos-7 cells 24 h after transfection. In the negative control, fluorescence emission was not detected (Additional file 1: Fig. S4).

### Induction of E2-specific antibodies in sera of immunized mice

**Fig. 2:**
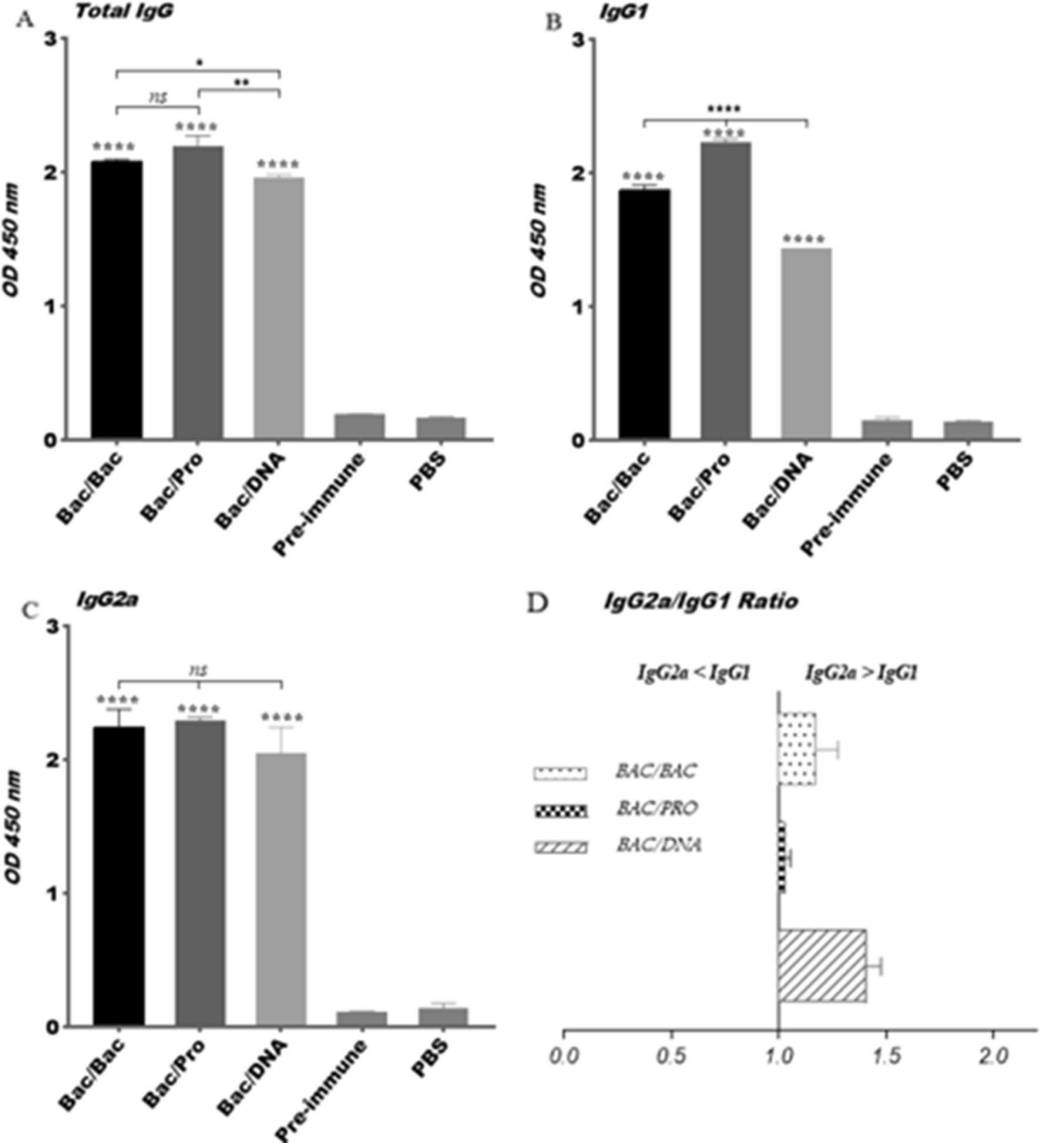
Antigen specific antibody responses induced in vaccinated groups. Vaccination strategies are shown in Table 1 and schematically indicated at the bottom of each graph. Sera samples were collected before immunization (pre-immune) and two weeks after the last immunization (week 8). Panel (**a**) shows specific anti-HCV-E2 total IgG levels, Panel (**b**) shows isotype IgG1 levels, panel (**c**) shows isotype IgG2a levels and Panel (**d**) shows IgG2a/IgG1 ratio. All samples were measured in duplicate. Comparison of ELISA results of each immunized group with controls (pre-immune sera and PBS group) was performed using unpaired t-test. Data are represented as mean ± SD. The asterisk indicates the significant difference between values determined by one-way ANOVA or Students t-test (*p* < 0.05 denoted as *, *p* < 0.01 denoted as **, *p* < 0.0001 denoted as **** and *ns* denoted as non-significant)

The level of rE2 specific antibodies including total IgG, IgG1 and IgG2a in the sera of immunized and control mice groups were evaluated by ELISA two weeks after the last immunization (week 8). As shown in Fig. 2, rE2 specific total IgG, IgG1 and IgG2a levels were significantly elicited in all vaccinated groups compared to the pre-immune sera and PBS control groups (*p* < 0.0001). The level of rE2-specific total IgG in Bac/Bac and Bac/Pro groups was significantly higher than the Bac/DNA group (*p* < 0.037, *p* < 0.002) (Fig. 2a). Also, immunization with Bac/DNA induced the lowest level of IgG1 and IgG2a compared to the Bac/Bac and Bac/Pro groups (Fig. 2b, c). However, there was significantly difference in IgG1 level between Bac/DNA with Bac/Bac and Bac/Pro groups (p < 0.0001). A significantly lower level of IgG1 was also observed in Bac/Bac group compared to the Bac/Pro (*p* < 0.0001) (Fig. 2c). The predominant IgG2a and IgG2a/IgG1 ratio > 1, suggested a shift toward Th1 immune responses for Bac/Bac and Bac/DNA groups (Fig. 2c, d). However, induction of similar levels of IgG1 and IgG2a in Bac/Pro mice group suggested a balanced Th1-Th2 phenotype (Fig. 2d). This may be due to the effect of Montanide ISA 720 used as adjuvant in combination with rE2 protein. Montanide ISA 720 is a water-in-oil emulsion that enhances immune responses by the depot effect and slow release of antigens at injection site. This adjuvant can also cause inflammation and stimulate the recruitment of antigen-presenting cells (APCs) such as macrophages and lymphocytes [38]. Previous studies have shown that Montanide ISA 720 induced strong and balanced Th1/Th2 responses [39].

### Bac/Bac immunization elicited the highest Th1-type cytokine response

**Fig. 3:**
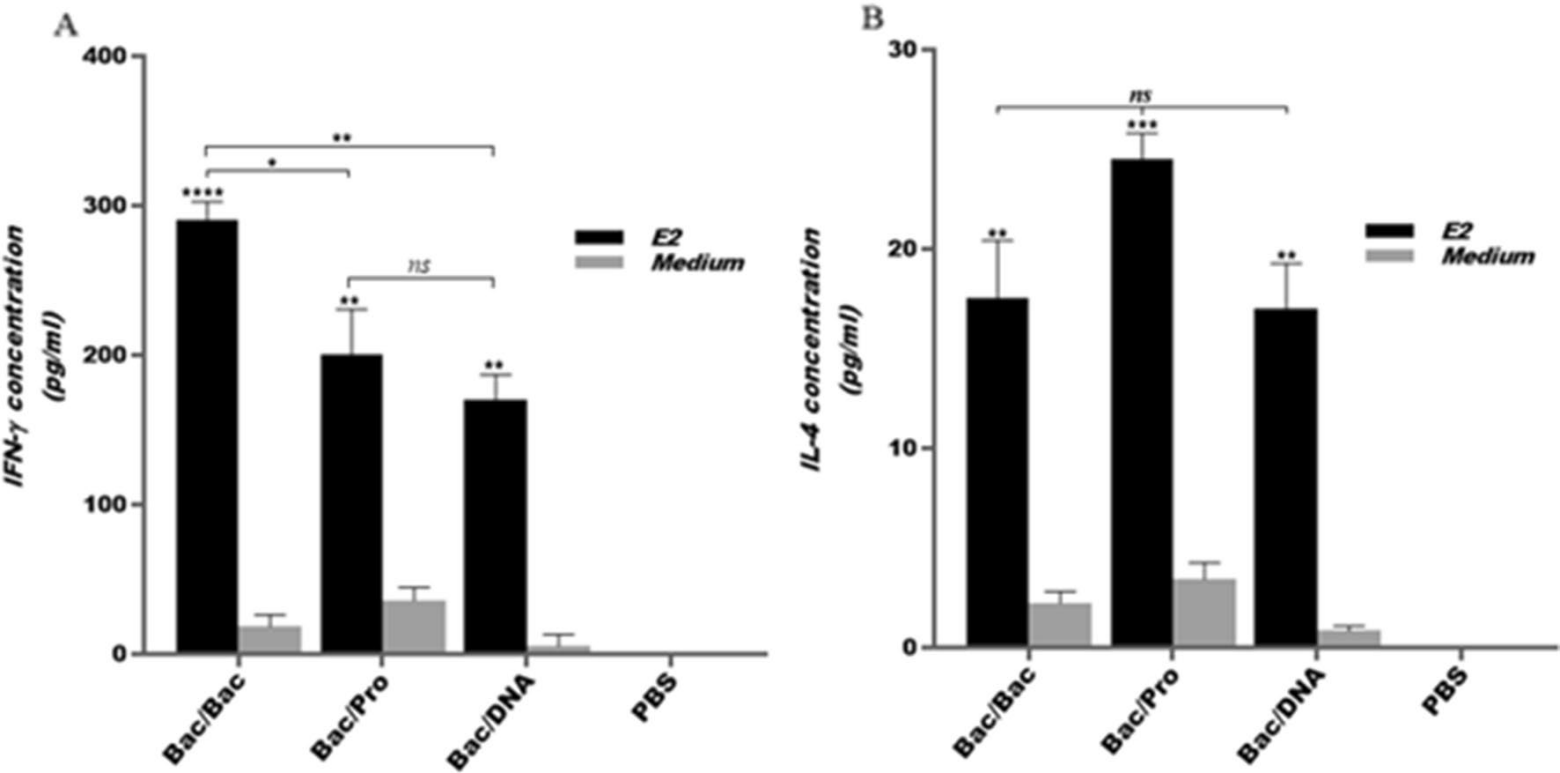
Cytokine levels in vaccinated mice groups. Cytokine production by splenocytes were evaluated 2 weeks after the last immunization. Splenocytes were cultured in duplicate and stimulated in vitro with HCV E2 protein (at 10 µg/ml), ConA as positive control (not shown) or medium alone (medium). IFN-γ (**a**) and IL-4 (**b**) levels in cell supernatants were measured by ELISA. The data are presented as the mean levels of cytokine ± standard deviation for 6 immunized mice in each group. One-way ANOVA was used for statistical analysis (*P* < 0.05 denoted as *, *P* < 0.01 denoted as **, *P* < 0.0001 denoted as **** and *ns* denoted as non-significant)

The level of Th1-associated cytokine IFN-γ was measured using Ag-stimulated splenocytes of all mice groups two weeks after the last immunization. All immunized mice groups showed a significant difference compared to the control group (PBS) (*p* < 0.0001, *p* < 0.01, Fig. 3). As shown in Fig. 3a, the Bac/Bac compared to the Bac/Pro and Bac/DNA induced significantly higher levels of IFN-γ production (*p* < 0.05, *p* < 0.01). However, the two groups Bac/Pro and Bac/DNA also indicated significant levels of this cytokine. We also examined whether immunized mice produced Th2-associated cytokine IL-4. All groups developed similar low levels (no significant differences) of this cytokine (Fig. 3b) indicating the shifted immune responses toward Th1 type polarization (the most potent for Bac/Bac group).

### Prime-boost regimen using Bac-CMV-E2-gp64 induced strong virus-cross-neutralizing antibodies

**Fig. 4:**
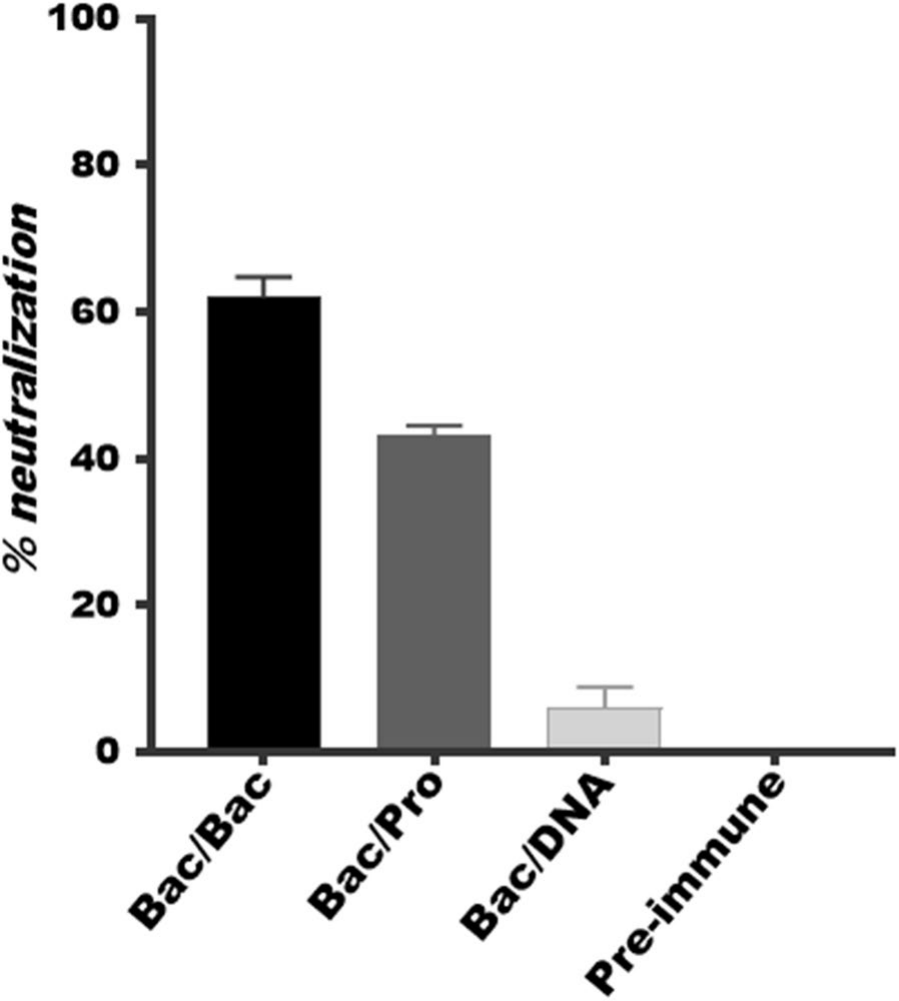
Induction of cross-neutralizing antibodies in immunized mice against HCVcc. Mice sera collected on day 0 and 56 were mixed at a 1:100 dilution with JFH-1/HCVcc and incubated for 1 h at 37 °C and subsequently applied to Huh-7 cells for 6 h in triplicate. Pre-immune sera was used as a negative control. The data are shown as the percent neutralization of JFH-1/HCVcc from sera of three mice groups (each had 8 mice) with different immunizations relative to the controls

The neutralizing capacity of the generated antibodies in the vaccinated mice against infectivity of JFH-1/HCV (genotype 2a) was analyzed. To this end, sera collected from all mice groups two weeks after the final immunization and that of the pre-immunized mice (mice sera collected on day 0; used as negative control) were used. The results are presented as percent cross-neutralization of virus infection compared to the pre-immune sera. The cross-neutralizing activities of Abs induced in response to immunization with Bac/Bac regimen (62%) were higher than those produced by immunization with Bac/Pro (43%), but much stronger than those elicited by immunization with Bac/DNA regimen (6%) (Fig. 4). Neutralization of JFH1-HCV (genotype 2a) by sera of mice immunized with a HCV E2 of 1a-subtype suggested that B cell responses might have been elicited towards conserved neutralizing epitope(s) in rE2 ectodomain. Neutralization was not observed for pre-immune sera while 100% neutralization was observed for AR5A as positive control.

## Discussion

In the present study, we reported the construction of a “BacMam virus-based surface display (Bac-CMV-E2-gp64)” that both expressed and displayed gpE2 in mammalian cells and bacouloviral envelope, respectively. Immunization studies with this live recombinant baculovirus in mice indicated that a homologous prime-boost vaccination regimen by this simple immunogen formulation (Bac/Bac) is superior than complicated heterologous immunization regimens with variable immunogen formulations (Bac/Pro and Bac/DNA) for induction of high levels of both NAbs and IFN-γ cytokine. Therefore, the constructed BacMam virus (Bac-CMV-E2-gp64) might be considered as a promising candidate to fulfill the demanded characteristics for induction of high levels of NAbs and potent T-cell responses for an efficient HCV vaccine.

As shown in Fig. 1a, use of the synthetic sequence of pCMV-SP-E2-TM-CTD for generation of BacMam virus-based surface display (Bac-CMV-E2-gp64) resulted the possibility of simultaneous expression (Fig. 1b) and display of HCV-E2 via translocation on SF9 cell membrane (Fig. 1c) and incorporation into baculovirus envelope through the budding process (Fig. 1d). Our results was in accordance with the prior reports indicated the efficiency of of BacMam virus-based surface display for dual expression and display of target antigens [24].

Results of the ELISA for HCV gpE2-specific IgG measurements on sera of the mice immunized by different immunization regimens indicated that there was statistically significant difference in the total IgG levels between Bac/Bac and Bac/Pro compared to Bac/DNA group (*p* < 0.017, *p* < 0.0007) (Fig. 2a). These results indicate that the first two groups might have more efficiently produced anti-E2 IgG levels which is in agreement with previous reports for positive effect of both the antigen displaying (by gp64 TM and CTD) or booster immunization with adjuvanted recombinant protein on enhancement of the humeral immune responses [40, 41]. Although in our study the difference of IgG levels between Bac/Bac and Bac/Pro groups was not detectable (Fig. 2a), but a prior heterologous prime-boost study with Ad6 encoding-E1E2/MF59 adjuvant + E1E2 immunogens in BALB/c mice reported significantly higher humoral immune responses than homologous prime-boost immunization by Ad6 encoding-E1E2 alone [42]. The reasons behind the efficiency of our Bac/Bac immunization regimen for induction of strong humeral responses might be proper presentation of the HCV gpE2 with natural conformation via display on the baculovirus envelope and the adjuvant activity of the vector due to the presence of CpG motifs within the baculoviral DNA genome, as well as, induction of lower baculovirus specific immunity compared to adenovirus vectors [25, 43]. In this context, frequency of the anti-baculovirus T cells compared to that of the anti-adenovirus in the similar immunogen formulations has been estimated to be less than 3.5 folds (140 SFU/5˟105 versus 500 SFU/5˟105 spelenocytes) [43, 44].

As shown in Figs. 2b, c and 3, measurement of the anti-E2 IgG isotypes and proinflamatory cytokines indicated higher levels of IgG2a induction compared to IgG1 (IgG2a/IgG1 > 1) and also high levels of IFN-γ compared to IL-4, for Bac/Bac and Bac/DNA groups. These results revealed a Th1 polarization of immune responses for both mice groups. Of note, compared with Bac/DNA, Bac/Bac group induced higher IgG1 and IgG2a antibody responses and also elicited significantly higher level of IFN-γ cytokine. The Th1-biased immune response is a demanded polarization for design of effective vaccines against HCV infection [45]. Based on our results, these characteristics might be accomplished by immunization via BacMam virus-based surface display for HCV gpE2 construct. Of note, a recent report on application of recombinant MVA vectors encoding HCV E1E2 (vvIV257) or ectodomains of E1 and E2 (vvIV316) or E2-RVGTM + E1-VSVGTM (vvIV306) for immunization indicated the elicitation of very low IgG2a and high levels of IgG1 and IgG2b towards a Th2 biased immune responses which imply the advantage of bacoulovirus as a vaccine modality for HCV gpE2 antigen [46]. In fact, the presence of CpG motifs within the baculoviral DNA genome (with significantly higher frequency than Ad5 and mammalian DNAs and also similar frequency to that of bacterial DNA) shows strong adjuvant properties on induction of immune responses in mammalian cells for production of pro-inflammatory cytokines that enhance the potentiate and the induction of both humoral and T cell responses [17, 47]. However, Bac/Pro group with high E2-specific Ab titers, notably of IgG1 and IgG2a isotypes induced antibody responses toward a mixed Th1-Th2 phenotype and calculation of the IFN-γ/ IL-4 ratio indicated this group induced these cytokine responses toward a Th1 profile. This result is in accordance with several prior reports on application of the BacMam virus-based surface display immunization strategies for various antigens in induction of mixed Th1/Th2 responses with Th1 polarization [21, 25].

As shown in Fig. 4, BacMam virus-based surface display for HCV gpE2 (Bac) in this study resulted to induction of cross-NAbs in the sera of all primed mice groups which was highest for the Bac/Bac group compared to Bac/Pro and Bac/DNA groups (62% versus 41% and 6%). Our results are consistent with a prior report on cross-neutralization of subtype-2a HCVcc (JFH1) by sera of the mice immunized by envelope proteins of HCV genotype 1a/strain H77 [41, 48]. These final results indicated several important propositions. First, since induction of NAbs needs the presentation of target protein in its native conformation, thus gpE2 is displayed in native form on the baculovirus surface, which can be evaluated by receptor- and antibody-binding ELISA assays in our future study [49, 50]. Second, induction of the highest humeral and cellular responses by Bac/Bac immunization regimen might be due to the dual expression system of BacMam virus-based surface display for both display of the target antigens on the viral envelope and their concurrent expression upon transduction in mammalian cells. Such dual functioning (expression/display) mimic the potential advantages of both DNA and subunit vaccines for presentation of the target antigen [19]. Third, induction of the high cross-neutralizing activity (neutralization of subtype-2a HCVcc (JFH-1) by homologous prime-boost Bac/Bac (encoding subtype-1a HCV gpE2) immunization regimen (62%) is a promising result for potential application of this system for design of an effective E2-encoding HCV vaccine with a simple, cost-effective and applicable formulation (identical prime/boost formulations) for the aim of population based immunization strategies.

In support of this proposition, it should be noted that several prior vaccine studies on HCV envelope antigens reported less percentages of neutralization by vaccine induced antibodies, in spite of using more complicated formulations and/or heterologous regimen of immunization which is not cost-effective and/or practical in population based immunization strategies [41, 48]. In this context, it was reported that homologous prime-boost immunization of mice with an HCV E1/E2 encoding alpha virus elicited 30% cross-NAb titers that could be only enhanced by 56% via heterologous boosting immunization with E1/E2 proteins formulated in MF59 + CpG adjuvants [48]. In another study, homologous prime-boost immunization of mice by measles virus-encoding HCV Core/E1/E2 (MV-CE1E2) or ectodomains of E1and E2 fused to MV F protein transmembrane domain (MV-E1/Ft-E2/Ft) elicited around 40% and 20% NAbs for homologous virus neutralization, respectively. Interestingly, in this prior study, boosting immunization with adjuvant-formulated rE2 protein in MV-primed animals resulted to cross-NAbs values of 34% and 4% for sera of the mice primed with MV-CE1E2 or MV-E1/Ft-E2/Ft, respectively [41]. However, it should be noted that the important shortcoming of this study similar to most of the prior studies, might be the limited evaluation of NAbs induced by " subtype 1a HCV-gpE2" against "subtype 2a (JFH-1) virus" that should be expanded for other HCV genotypes in future studies. Recently, results of immunization by a vaccine formulation based on sE2-ferritin nanoparticles indicated the induction of high cross-neutralizing activity (from 65 to 95% depending on the used E2 dosage) [50]. It should be noted that this high neutralizing activity (which seems to be higher than that of our study) might be not only due to the use of the ferritin nanoparticles as platform but in part due to the genotype by which E2 was coded, dilution of antiserum sample used for the test and numbers of vaccine injection, too.

Overall, comparing with prior reports, Bac/Bac immunization approach in the present study, elicited strong immune responses that are suggested for correlation with protection from HCV infection including high level cross-NAbs and potent cellular responses [51–54]. The reasons behind these promising results might be the intrinsic adjuvant properties of bacoulovirus in general and BacMam virus-based surface display in specific for acting simultaneously as both subunit and DNA vaccines [19]. Indeed, based on prior suggestions, it might be proposed that HCV gpE2 displayed by our designed BacMam virus was internalized and processed by APCs and most likely presented through the MHC II pathway to stimulate CD4^+^ helper T cells which is critical for effective B cell response and generation of antiviral cytokines [11, 23]. Although not investigated in this study, it is most likely that CD8^+^ T cell responses against gpE2 could also be induced via MHC I pathway by the CMV promoter-based expression of gpE2 in transduced cells [20, 25]. Understanding the capability of this constructed BacMam virus as HCV vaccine in induction of a potent CD8 + T cell responses will be the focus of our future work.

## Conclusions

To summarize, in the present study, for the first time to our knowledge, we reported the construction of a BacMam virus-based surface display for HCV gpE2 and provided evidence for simultaneous expression and display of the gpE2 in mammalian cells and bacoulovirus envelope, respectively. Results of animal vaccination by the constructed Bac-immunogen in homologous prime/boost immunization indicated HCV E2-specific, strong humeral and cellular responses. The Bac/Bac immunization induced cross-reactive NAbs with percentages of viral neutralization comparable with (higher than) the prior reports on application of the same antigen in other viral vectors as HCV candidate vaccines. This promising result sounds for potentiality of BacMam virus-based surface display in construction of an effective HCV vaccine.

## Supplementary Information

**Additional file 1: Supplemental Table 1.** DNA sequence of the used primer. **Figure S1.** Schematic representation for plasmid construction of the recombinent Bac-E2/exp and production of HCV gpE2 protein in insect cells. **Figure S2.** Schematic representation for construction of the pEGFP-E2-NT(gp96) vector and its expression in COS-7 cell line. **Figure S3.** Schematic representation for construction of the pCDNA-E2-NT(gp96) vector. **Figure S4.** Analysis of the *In vitro* expression of pEGFP-E2-NT(gp96), in COS-7 cells by fluorescence microscopy.

## Data Availability

The datasets used and/or analyzed during the current study are available from the corresponding author on reasonable request.

## Abbreviations

HCV: Hepatitis C virus
DAAs: Direct-acting antivirals
NAbs: Neutralizing antibodies
IFN: Interferon
JFH-1: Japanese fulminant hepatitis 1 strain
gpE2: Envelope E2 glycoprotein
PCMV-IE: Immediate early cytomegalovirus promoter
MHC: Major histocompatibility
SP: Signal sequence
TM: Transmembrane
CTD: Cytoplasmic terminal domain
Sf9: Spodoptera frugiperda
MOI: Multiplicity of infection
NT(gp96): N-terminal region of gp96 gene
MAb: Monoclonal antibody
MVA: Modified vaccinia virus Ankara
APC: Antigen presenting cell
HCVcc: Cell culture derived HCV particles
ConA: Concanavalin A
