# Supplementary material for: BacMam virus-based surface display for HCV E2 glycoprotein induces strong cross-neutralizing antibodies and cellular immune responses in vaccinated mice
Source: Infect Agent Cancer. 2021 Dec 18;16:69. doi: 10.1186/s13027-021-00407-x (PMC8684228; doi:10.1186/s13027-021-00407-x)
Supplement: Supplementary file 1 — Additional file 1: Supplemental Table 1. DNA sequence of the used primer. Figure S1. Schematic representation for plasmid construction of the recombinent Bac-E2/exp and production of HCV gpE2 protein in insect cells. Figure S2. Schematic representation for construction of the pEGFP-E2-NT(gp96) vector and its expression in COS-7 cell line. Figure S3. Schematic representation for construction of the pCDNA-E2-NT(gp96) vector. Figure S4. Analysis of the In vitro expression of pEGFP-E2-NT(gp96), in COS-7 cells by fluorescence microscopy. [file 13027_2021_407_MOESM1_ESM.docx]

**Supplementary figures**


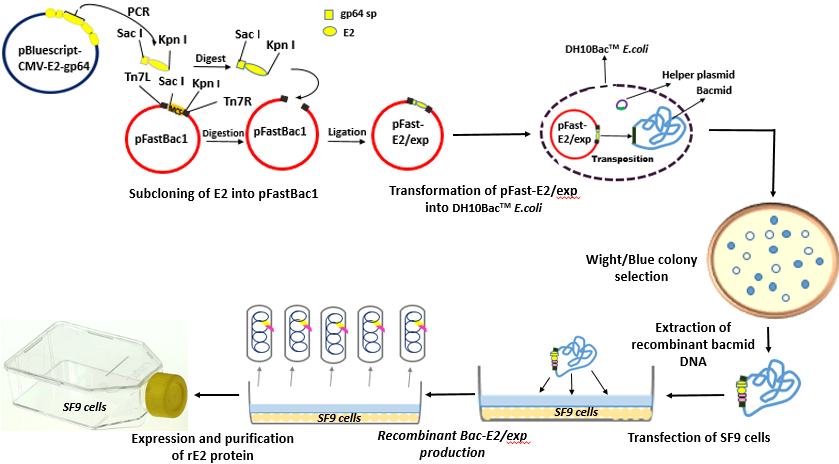


**Supplementary Fig. S1** Schematic representation for plasmid construction of the recombinant Bac-E2/exp and production of HCV gpE2 protein in insect cells. The pBluescript-pCMV-E2-gp64 vector was constructed by insertion of the synthesized fragment (pCMV-SP-E2-TM-CTD) into the Xho I and Not I sites of the vector (Biomatik Corporation, Canada) was used as template for construction of Bac-E2/exp vector. This vector encoded for immediate early cytomegalovirus promoter (PCMV-IE), the signal sequence of gp64 (the major envelope fusion glycoprotein of baculovirus) (SP), a stretch of six histidine residues (His6 tag), the ectodomain of HCV E2 protein (E2, codon 384-661, genotype 1a, Gen Bank Accession No: AF011753.1) and the transmembrane (TM) and the cytoplasmic terminal domain (CTD) of gp64 in tandem. As shown in Fig. S1, the SP(gp64)-His6-E2 fragment was PCR-amplified by using pFast-F and pFast-R primers (Supplementary Table S1). The primers were designed to add restriction enzyme sites, the *Sac* I and *Kpn* I to the 3´ and 5´terminal of the PCR products, respectively. The E2 amplicon was cloned into the *Sac* I and *Kpn* I sites of the transfer vector pFastBac1 (Invitrogen, Carlsbad, CA, USA) to construct pFast-E2/exp. Subsequently, the recombinant transfer plasmid pFast-E2/exp was transformed into the competent DH10Bac^TM^ *E.coli* cells. Due to the presence of site-specific transposons on pFastBac I plasmid (Tn7L and Tn7R) and Tn7 transposition function of helper plasmid, the E2 construct integrate into the baculovirus shuttle vector (Bacmid) through site specific transposition. The resulting recombinant bacmids were isolated from white colonies via whit/blue colony selection. Following the confirmation of the target gene fragment by PCR using universal M13 (pUC/M13-F and pUC/M13-R) primers (Supplementary Table S1), the recombinant Bac-E2/exp was generated by transfection of recombinant bacmid DNA into the *Spodoptera frogiperda* (Sf9) cell lines (ATCC, USA) using the Bac-To-Bac Baculovirus Expression System (Invitrogen, Carlsbad, CA, USA). Briefly, Sf9 cells were transfected with recombinant bacmid DNA mixed with 8 µl cellfectin II (Invitrogen, USA) in unsupplemented Grace’s Insect Cell Culture Medium. The supernatant containing released virus particles harvested 96 h post transfection, centrifuged, and used for more amplification of the viruses to reach final titer of 1× 10^8^ pfu/ml. The viral particles were purified by two rounds of sucrose gradient ultracentrifugation as described previously [1]. The purified recombinant baculoviruses were resuspended in phosphate-buffered saline (PBS). The infectious titers of the recombinant bacoloviruses were determined by plaque assay. For expression and purification of recombinant E2, the Sf9 cells (1×10^7^) were infected with recombinant baculovirus Bac-E2/exp at a MOI of 10 and incubated at 27°C for 48 hours. The culture supernatant was centrifuged at 500 g for 10 min and dialysed against the buffer (100 Mm Tris-HCL pH 8.0, 100Mm Nacl and 5 mM Mgcl2). Recombinant E2 (rE2) was purified by affinity chromatography using 6x His-tag on a Ni-NTA super flow column according to the manufacturer´s protocol (The QIA expressionist ^TM^, QIAGEN, Valencia, CA). The rE2 protein was eluted with 250 mM imidazole in dialysis buffer. The purity of the protein was analyzed by 10% SDS-PAGE. Protein concentration was determined using BCA assay Kit (Pierce, Thermoscientific). The purified rE2 protein was used as boosting immunogen (in combination with montanide adjuvant) in mice immunization procedures in indicated groups (see methods section of the manuscript) and as coating antigen for enzyme-linked immunosorbent assay (ELISA) and cytokine assays. Constructed vectors were confirmed by restriction analysis and DNA sequencing reactions. Molecular cloning procedures were based on routine protocols and/or manufacturer recommendations [2].


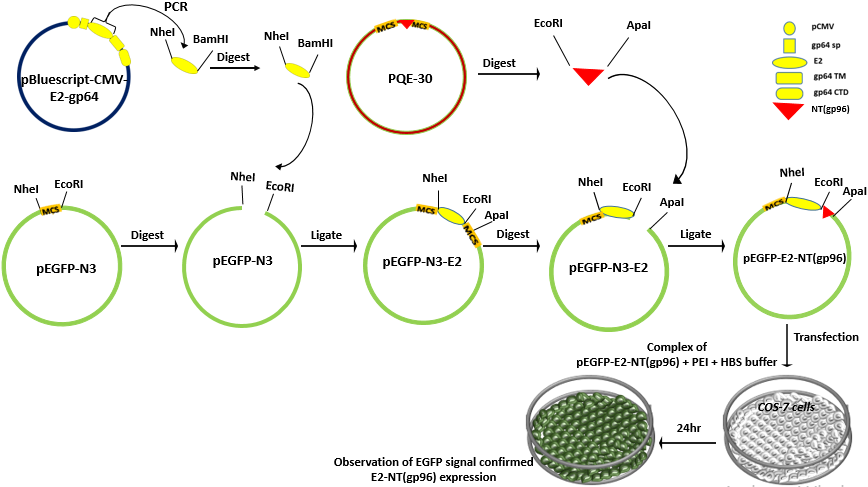


**Supplementary Fig. S2** Schematic representation for construction of the pEGFP-E2-NT(gp96) vector and its expression in COS-7 cell line. To generate the pEGFP-E2-NT(gp96) vector, first the pEGFP-E2 plasmid was constructed by excision of the E2 gene from pBluescript-CMV-E2-gp64 by digestion with *Nhe* I and *EcoR*I enzymes and ligation into the same sites of pEGFP-N3 plasmid (clontech, USA). Subsequently, the gene fragment encoding for the N-terminal domain of the gp96 (NT(gp96)) from pQE-30-gp96 vector was excised by *EcoR*I and *Apa* I restriction enzymes. The excised fragment "NT(gp96)" was cloned into the *EcoR*I and *Apa* I sites of the pEGFP-E2 plasmid. The pQE-30-gp96 harboring the NT(gp96), Xenopus Laevis, GenBank accession No. AY187545.1) was described previously [3]. To demonstrate *in vitro* expression of pEGFP-E2-NT(gp96) in COS-7 cell line, up to 5×10^4^ COS-7 cells per well of a four well plate (Greiner, Germany) was incubated until the cells reached a confluence of 75%.  *In vitro* expression of pEGFP-E2-NT(gp96) in COS-7 cell line was evaluated by transfection of these cells with 5 µg of pEGFP-E2-NT(gp96), pEGFP-N3 (positive control) and pcDNA3.1 (negative control) plasmids in separate reactions using LINPEI 25KDa (10 μM; poly sciences, Europe) as described previously [4]. Expression of the proteins was confirmed by observation of the EGFP signal under fluorescence microscope (Nikon E200, USA) at 24 h post transfection. Constructed vectors were confirmed by restriction analysis and DNA sequencing reactions. Molecular cloning procedures were based on routine protocols and/or manufacturer recommendations [2]**.**

**
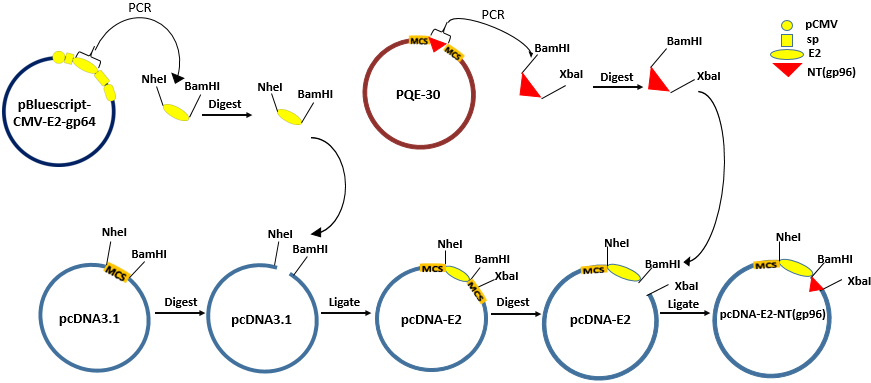
**

**Supplementary Fig. S3** Schematic representation for construction of the pCDNA-E2-NT (gp96) vector. To generate pcDNA-E2-NT(gp96) plasmid, the HCV E2 ectodomain was amplified from the pBluescript-CMV-E2-gp64 vector using the PC-E2-F and PC-E2-R primers (Supplementary Table S1). The primers were designed to add *Nhe* I and *BamH* I restriction sites, to the head and tail of the the amplified fragments, respectively. The PCR-amplified fragment was cloned into the same digested sites (*Nhe* I/*BamH*I) of pcDNA3.1(+) vector (Invitrogen, Carlsbad, CA) to generate pcDNA-E2. Subsequently, the N-terminal domain of the gp96 (NT(gp96)) was amplified from pQE-30-gp96 vector using the PC-gp96-F and PC-gp96-R primers (Supplementary Table S1). The primers were designed to generate targeted restriction sites (*BamH* I and *Xba* I) in the amplified fragments. The PCR-amplified NT(gp96) gene was cloned into the *BamH*I/*Xba I* sites of the recombinant pcDNA-E2 to generate pcDNA-E2-NT(gp96) plasmid. The pcDNA-E2-NT(gp96) plasmid was purified by ion-exchange chromatography with Endo Free plasmid Giga Kit (QIAGEN, Valencia, CA) and used as boosting immunogen in mice immunization procedures in the indicated groups (see methods section of the manuscript). Precision and accuracy of the constructs were confirmed by restriction analyzing and DNA sequencing. Molecular cloning procedures were based on routine protocols and/or manufacturer recommendations [2].





**Supplementary Fig. S4** Analysis of the *In vitro* expression of pEGFP-E2-NT(gp96), in Cos-7 cells by fluorescence microscopy. Recombinant pEGFP-E2-NT(gp96), pEGFP-N3 (as positive control) and pcDNA3.1 (as negative control) complexed with polyethylenimine were transfected into Cos-7 cells. Expression of the proteins was confirmed by observation of the EGFP signal under fluorescence microscope (Nikon E200, USA) at 24 h post transfection. (A) and (B) GFP expression of transfected cells (before and after glinting of fluorescence) with pEGFP-N3 as positive control and pEGFP-E2-NT(gp96), respectively. No fluorescence emission could be recovered from cos-7 cells transfected with pcDNA3.1 plasmid as negative control (data not shown).

**Supplementary table**

**Supplementary Table S1** DNA sequence of the used Primers.

| **Primers** | **Sequences** |
| --- | --- |
| pUC/M13- F | 5′-CCCAGTCACGACGTTGTAAAACG-3′ |
| pUC/M13-R | 5′-AGCGGATAACAATTTCACACAGG-3′ |
| Bac-E2-F | 5′-ATCACCATCACGAAACCCAC-3′ |
| Bac-E2-R  pFast-F  pFast-R  PC-E2-F  PC-E2-R  PC-gp96-F  PC-gp96-R | 5′-CGTGCTATTGATGTGCCAAC-3′  5′- TAGCGAGCTCACCATGGGCCTACTAGTAAAT-3′  5′-CATGAGGTACCTTACTCGGACCTGTCCCT-3′  5′-GGCGCTAGCATGCTACTAGTAAATCAGTCAC-3′  5′-TAGGATCCCTCGGACCTGTCCCTGTCTTC-3′  5′-ATATGGATCCGAAGATGACGTTG-3′  5′-GGGCTCTCTAGATTATTTGTAGAAGGCTTTG-3′ |

pUC/M13-F/R and Bac-E2-F/R pair were used to confirm the insertion of target gene.

pFast-F/R pair amplified SP-E2 to construct pFast-E2/exp and inserted the SacI and *KpnI* (underlined) restriction enzymes sites, respectively.

PC-E2-F/R pair amplified E2 to construct pcDNA-E2 and inserted the NheI and *BamHI* (underlined) restriction enzymes sites, respectively.

PC-gp96-F/R pair amplified NT(gp96) to construct pcDNA-E2-NT(gp96) and inserted the *BamHI* and *XbaI* (underlined) restriction enzymes sites, respectively.

**References**

1. O’Reilly, D., Miller, L.K., Luckow, V. A laboratory manual. New York: Oxford University Press; 1992.

2. Sambrook, J., Russell, D., 2006. The Condensed Protocols From Molecular Cloning: A Laboratory Manual Author: Joseph Sambrook, David W. Russell, Publisher.

3. Pishraft-Sabet, L., Kosinska, A.D., Rafati, S., Bolhassani, A., Taheri, T., Memarnejadian, A., Alavian, S.-M., Roggendorf, M., Samimi-Rad, K., 2015. Enhancement of HCV polytope DNA vaccine efficacy by fusion to an N-terminal fragment of heat shock protein gp96. Archives of virology 160, 141-52.

4. Sabet, L.P., Taheri, T., Memarnejadian, A., Azad, T.M., Asgari, F., Rahimnia, R., Alavian, S.M., Rafati, S., Rad, K.S.J.H.m., 2014. Immunogenicity of multi-epitope DNA and peptide vaccine candidates based on core, E2, NS3 and NS5B HCV epitopes in BALB/c mice. Hepat Mon 14.
